# Combining Social Norms and Social Marketing to Address Underage Drinking: Development and Process Evaluation of a Whole-of-Community Intervention

**DOI:** 10.1371/journal.pone.0169872

**Published:** 2017-01-20

**Authors:** Sandra C. Jones, Kelly Andrews, Kate Francis

**Affiliations:** Centre for Health and Social Research, Australian Catholic University, Melbourne, Victoria, Australia; Leibniz Institute for Prvention Research and Epidemiology BIPS, GERMANY

## Abstract

Youth alcohol consumption has been steadily declining in Australia, as in other countries; fewer young people are drinking and the age of initiation is increasing. However, young people, their parents and others in their communities continue to believe that adolescent (excessive) drinking is the norm. This perception, and the concurrent misperception that the majority of parents are happy to provide their underage children with alcohol, creates a perceived culture of acceptance of youth alcohol consumption. Young people believe that it is accepted, and even expected, that they will drink; and parents perceive that not providing their adolescent children with alcohol will lead to social exclusion. There is evidence that shifting social norms can have an immediate and lasting effect adolescents’ (and adults’) alcohol related attitudes and behaviors. This paper reports on a novel, community based social marketing intervention designed to correct misperceptions of alcohol related social norms in an Australian community. The project utilized a social marketing approach, informed by the full complement of Andreasen’s social marketing benchmarking criteria, and concurrently targeted adolescents, parents of adolescents and the broader community. Using extensive formative research and multiple evaluation techniques, the study demonstrates that shifts in community social norms are possible and suggests that this approach could be used more widely to support the positive trends in youth alcohol consumption and parental supply.

## Introduction

Underage drinking is an issue of concern around the world. In 2011, 51% of Australian secondary school students reported consuming alcohol in the last year; 34% of 15-year-old, 48% of 16-year-old and 59% of 17-year-old Australians had consumed alcohol in the last month [1] (compared to 11% of US 14–15 year olds and 25% of US 16–17 year olds [2]). However, the 51% who consumed alcohol in 2011 represents a substantial decrease from previous years (down from 61% in 2008 [3]); and studies show Australian teens are initiating drinking later and the proportion abstaining is increasing [4].

Unfortunately, the majority of teens over-estimate peer alcohol consumption and believe underage drinking is an accepted and expected behavior [5–7]. The perceived normative nature of underage alcohol consumption is further exacerbated by, and evidenced by, the provision of alcohol to young people by parents and other responsible adults; in the 2011 Australian secondary school survey, 33% of current drinkers (those who had consumed alcohol in the last week) received their last drink from their parents, 23% from friends, and 21% from someone else (presumably an adult) who purchased it for them; only 10% took the alcohol from home or purchased it themselves [1].

The social norms approach (SNA) has been shown to be a powerful approach to behavior change. SNA is consistent with substantial evidence that people are motivated to conform to the behavior of others [8]; thus, we can change people’s behavior by correcting their misperceptions of what is normative. The SNA has been used successfully to reduce alcohol consumption in other target groups, including US university students [9–11]. While less extensively researched, it appears SNA can be successful in reducing alcohol beliefs and intentions among younger adolescents [9, 12, 13]. It is also likely that such approaches could be successful with parents and other adults, who perceive their own attitudes to the provision of alcohol to minors to be more conservative than those of other adults in their community [14].

There is increasing evidence that social marketing (SM) approaches can produce positive changes in drinking behaviors. A 2007 review noted several studies showed significant short-term effects and two showed some effect over two years [15]. A more recent review of 23 social marketing interventions targeting alcohol related harm reported that 13 of the 16 that reported on behavioral outcomes identified at least one positive outcome [16]. In both reviews, the successful interventions tended to be those that incorporated all or most of the elements of social marketing rather than uni-dimensional ‘education’ or ‘communication’ campaigns.

It is important to note that ‘social marketing’ and ‘social norms’ are not interchangeable, but the two concepts may be able to be effectively combined. Social norms is a specific theoretical approach, focused on correcting misconceptions concerning the prevalence of a particular behavior; social marketing is a framework for influencing behavior through the use of marketing principles [17]. It has been suggested that the social norms approach has been underutilized in social marketing and that social marketing could learn from, and contribute to, the evidence base in SNA [18]. For example, the SNA literature demonstrates the importance of using appropriate reference groups, incorporating credible data, and supporting advertising campaigns with more detailed information and resources.

## Method—The Social Marketing Program

The project reported in this paper aims to reduce the perceived normative nature of underage drinking and supply of alcohol to minors and, in the longer-term, reduce alcohol consumption among young people aged 12–17 years. The intervention is novel in that takes a community-based social norms approach, within a social marketing framework (see Table 1) and is further characterized by a staged research planning process consisting of formative, pretest, monitoring and evaluative research methods. The Kiama ‘Stop Underage Drinking Project’ is a comprehensive intervention that concurrently targets, adolescents, parents and the broader community.

**Table 1:** The Kiama Underage Drinking Project—Social Marketing Approach.

Andreasen’s six benchmark criteria		The Intervention
1. Behavior change	The intervention seeks to change behavior and has specific measurable behavioral objectives	While our overarching aim was not to change current drinking behavior, we sought to change the underlying beliefs and norms that will drive this behavior into the future; the changes in perceptions of community acceptance of underage drinking and alcohol supply, along with the increase in the age at which drinking initiation is deemed acceptable, shows that this approach will impact on future behaviors (not encouraging early initiation) as well as supporting and sustaining current positive behaviors (normalizing not drinking and not providing alcohol).
2. Consumer research	Formative research is conducted to identify consumer characteristics and needs. Interventions are pre-tested with the target group	The project was grounded in a strong and enduring consumer orientation—underpinned by extensive formative research and the guidance of a Community Consultative Committee throughout the two year campaign. *Consumer insights* not only guided the overall direction of the campaign (such as the use of a staged approach, meeting the community where is was by commencing with fear based messaging and then progressing with strong efficacy based and positive social norms messages) but also specific execution elements (such as the tagline for phase one being a direct quote from a focus group participant).
3. Segmentation and targeting	Different segmentation variables are used and a strategy tailored to the segments	The *segmentation* approach enabled us to develop and target our messages; beyond the initial geographic segmentation to the development of specific messages and strategies for three distinct target groups (12-to-17-year-olds, parents of 12-to-17-year-olds, and the broader community).
4. Marketing mix	The intervention must consist of communications plus at least one other ‘P’	All four Ps of the *marketing mix* were given equal consideration and emphasis in the development and implementation of the campaign. P*roduct* was awareness and acceptance of the actual norm that most teens don’t drink and most adults do not facilitate (or condone) underage drinking. P*rice* included deeply-held misperceptions of descriptive and injunctive norms. P*lace* was integral to the whole-of-community approached with saturation of messages and messengers, in partnership with a range of community partners including Kiama High School, Kiama Youth Centre, North Kiama Neighbourhood Centre, local clubs, local media and NSW Police. P*romotional* activities (paid and unpaid media, merchandise etc) were developed in collaboration with the community and were constantly refreshed to avoid message wear-out.
5. Exchange	The intervention considers what will motivate people to engage voluntarily with the intervention and offers them something beneficial in return, whether that is intangible or tangible	The principle of *exchange* ensured that we focused on building self-efficacy and addressing crucial barriers to engagement such as ensuring a sense of community ownership and that young people and parents felt supported, rather than criticized, by the intervention.
6. Competition	The intervention considers the appeal of competing behaviors (including the current behavior) and uses strategies to decrease competition	The intervention tackled the *competition* head on, by providing all three target segments with clear and accessible information on the realities of youth drinking, integrating into school curriculum opportunities for adolescents to discuss and develop viable alternatives to drinking, and developing messages and strategies that addressed all segments’ fears of being branded a ‘wowser’ in a pro-alcohol culture

### Formative Research

The first phase of the formative research was designed to explore currently held beliefs and attitudes in the community. All study protocols were approved by the University of Wollongong Human Research Ethics Committee (HE13 081). Participants for qualitative methods confirmed that they had read the information sheet and provided written informed consent. Participants for CATI survey provided informed verbal consent. Data was de-identified prior to analysis. The formative research consisted of six interviews with key stakeholders (two local police officers, two general practice doctors, a secondary school teacher and a youth worker); seven focus groups with teenagers; four focus groups with parents; and three focus groups with community members. Due to the sensitive nature of the topic for parents (provision of alcohol to their underage children), we also conducted three anonymous telephone interviews with parents and an anonymous online survey using a projective methodology (n = 180). The results of the formative research were used to develop a design brief for the development of three message concepts for testing with the target audiences. This testing consisted of focus groups (seven with adolescents, five with parents, and three with community members) and an anonymous online survey (n = 154). In addition to assessing responses to the proposed messages, the focus groups were used to explore with the three target audiences perceived acceptability of a range of intervention components.

The data consistently reflected a general consensus among all three target groups that the majority of teenagers (including those in their town) are drinkers, despite consistent evidence from national surveys that this is not the case. Additionally, the data revealed a strong belief that the primary driver behind youth drinking was the perceived need to fit in with peers (the social norm). The majority of younger teens reported that their parents would not let them ‘drink’ but would let them have small amounts of alcohol at home; and the older teens that their parents would let them drink in ‘safe’ situations. Parents’ reports were generally consistent with this—while all agreed that neither they nor their friends would let their children ‘drink,’ many said they would allow them to consume small amounts of alcohol at home or in ‘safe’ environments. Community members agreed that it was normative to provide alcohol to young people, and the majority said that they would probably do so. However, all three groups were critical of ‘bad’ kids who ‘drink’ and ‘bad’ parents who provide them with alcohol. Consistent with this, teens and parents expressed a preference for high-fear messages that targeted ‘those’ kids and parents and sought to address ‘their’ problematic drinking and alcohol provision. The projective study found the importance of children ‘fitting in’ with peers was the primary perceived motivator for both the mother and the father providing alcohol [19]. This suggests some parents may perceive the risks of alcohol-related harm to be the lesser evil compared to the social isolation of not fitting in with peers.

### Intervention Development and Implementation

The formative research identified that most parents perceived the ‘problem’ of adolescent alcohol consumption to be that affected ‘other’ families or from ‘other’ communities (not the children from ‘normal’, functional families such as theirs). These perceptions are consistent findings from previous studies regarding the influence of injunctive norms on parental attitudes and decision making (e.g.: [20]). They also go some way to explaining their preference for fear based messages for the ‘other’, with the Third Person Effect suggesting that such messages are perceived to have greater relevance and effectiveness for others than oneself [21].

**Fig 1:**
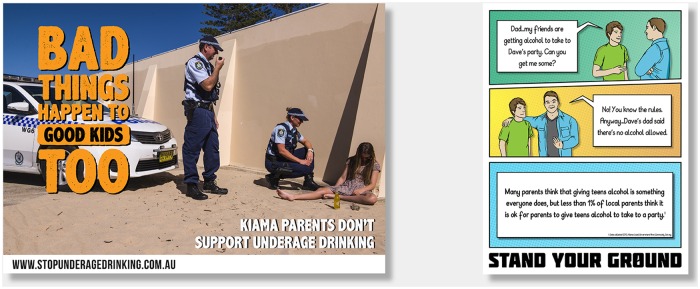
Examples of parent-targeted messages.

Thus, the parent component included posters and other print resources designed to raise the salience of the message, and ensure that parents were aware that they (and their children) were the target audience. A direct quote from a parent participant of the formative research provided the tagline, “Bad things happen to good kids too”. The parent intervention also included parenting workshops (addressing issues such as how to talk to teens about drinking), parent information sessions at school events, mailouts of information via the school, and a parent-targeted section on the website. See Fig 1 for examples of the parent-targeted messages.

**Fig 2:**
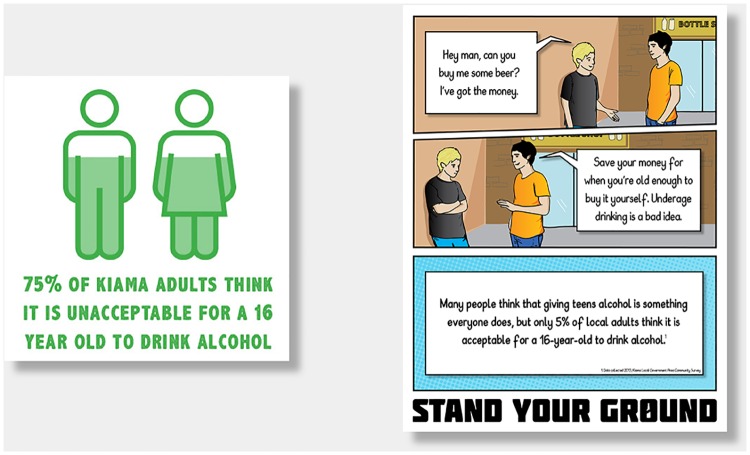
Examples of community-targeted messages.

The formative research identified that many community members perceived the ‘problem’ of adolescent alcohol consumption to be the result of a more ‘relaxed’ style of parenting and lack of discipline; similar perceptions of parental liability have been found internationally (e.g.: [22]). However, they also reluctantly acknowledged that negative consequences of underage drinking could happen to anyone. Thus, the community-wide components were designed to raise the salience of underage drinking as a community concern and a community responsibility. “Kiama Community Doesn’t Support Underage Drinking” was featured as an overarching tagline to most materials. In addition to the poster campaign and a community section on the website, key components included news and editorial items as well as advertising in the local newspaper; sticker placement on wheelie bins (household garbage cans) and a Facebook page. The project team was highly visible in the community, attending all major community events (such as festivals and sporting events) as well as the monthly market days; distributing information and project merchandise (e.g., pens, carry bags, and magnets with campaign messages). See Fig 2 for examples of the community-targeted messages.

**Fig 3:**
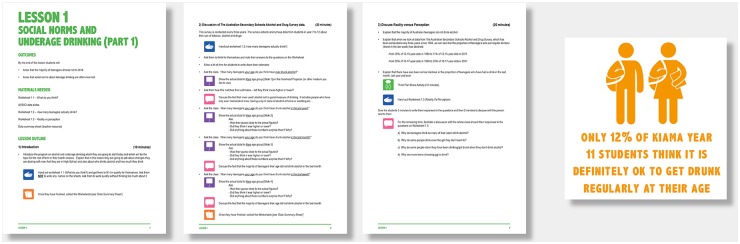
Examples of adolescent-targeted messages.

The intervention also targeted adolescents via a comprehensive, one term (10-week) social norms curriculum delivered to all students in the local high school in years seven through ten (aged 12 to 16 years), information sessions and presentations, a teen section on the intervention website with downloadable information and resources, a poster competition, and giveaways (such as hacky sacks, wristbands, and compact mirrors). See Fig 3 for examples of the adolescent-targeted messages.

Phase 1 (launched in Oct 2013) was designed to introduce the issue of underage drinking as a matter of concern, and responsibility, for all members of the community. We used a tagline based on a comment from one of our focus group participants (“Bad things happen to good kids too”) and the images featured scenes and locations that were relevant to the community (such as members of the local police team looking after a drunken teen on the local beach). Phase 2 (launched in Jan 2014) provided the community with a call to action, “Can a community stop underage drinking?” in a six week ‘teaser’ campaign, followed by the addition of “Kiama can” on all communication materials (also six weeks) and included customisable posters and banners to enable local groups to express their support for the campaign. Consistent with the literature on SNA [19] [19], Phase 3 (launched in April 2014) focused on normative behavior within the local community and provided strong social norms messages predominantly through a poster campaign, supported by a variety of media and marketing activities. Messaging consistently referred to ‘our community’ reinforcing normative perceptions of drinking among those in the Kiama community.

### Pretest

A CATI survey of adults in the targeted regional community was conducted prior to the development of the intervention to explore their perceptions of the acceptability of consumption of alcohol by adolescents under the legal alcohol purchase age, and provision of alcohol to adolescents by their parents and other adults. A commercial provider was contracted to conduct the survey using a computer-aided telephone interviewing (CATI) system. The selection criteria were residents of the relevant community who had lived there for 6 months or longer and were aged 18 years or older. Quotas were established to ensure an approximately even number of male and female respondents, and approximately 50% with dependent children. The sample base for the survey was the electronic White Pages. Due to the known churn of telephone numbers and the increasing proportion of silent numbers, the provider used a technique that starts with the population of numbers listed in the telephone book and adds new and unlisted numbers. Once the potential universe of numbers had been generated, a computer program was used to randomize the database and a sequential sample was extracted. The sample was geographically stratified and evenly distributed within strata. This process gave an even distribution of potential numbers across the target community. At the end of the survey respondents were asked whether they would be willing to be contacted again in 12 months. Key findings included over-estimations of the proportion of adolescents who are drinkers, and the perception that the general community’s attitudes towards underage drinking and the provision of alcohol to minors are more liberal than their own [14]. When asked “what percentage of 16 year olds consume alcohol”, the mean response was 53%; the reality is that while 48% of 16-year-olds report consuming any alcohol in the last month, only 29% are classified as current drinkers [1]. Respondents were asked a series of six questions with regard to their views of the acceptability of various alcohol supply behaviors (by parents or other adults; at home or away from home; supervised or unsupervised), and the same series in regard to their perception of the views of their community. Responses were on a five-point scale and participants responded to the same six statements from their own perspective and that of what they believed other adults in the community thought. Respondents perceived the community to be more accepting of all six underage alcohol supply behaviors than themselves; the average difference in “unacceptable” rating for the 6 items was 16.8% (range 11.3% to 29.5%).

### Process/Monitoring Evaluation Planning

To effectively understand how, or if, implementation strategies and activities were working towards the program objectives, a comprehensive process-evaluation plan was established and consistently revised; reflecting both the iterative nature of working within complex systems of community as well as providing both formative and summative data [23].

**Fig 4:**
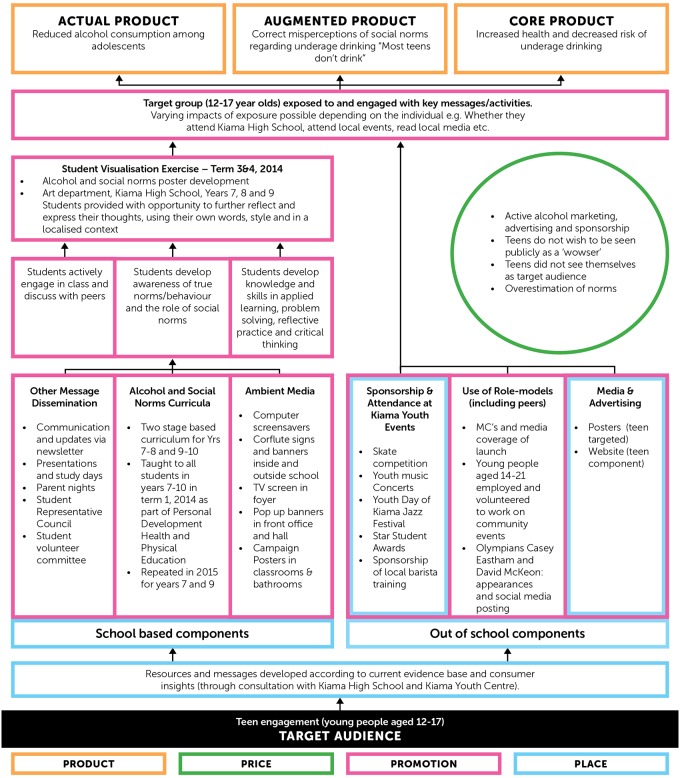
Project Logic Model—Adolescent Target Audience.

**Fig 5:**
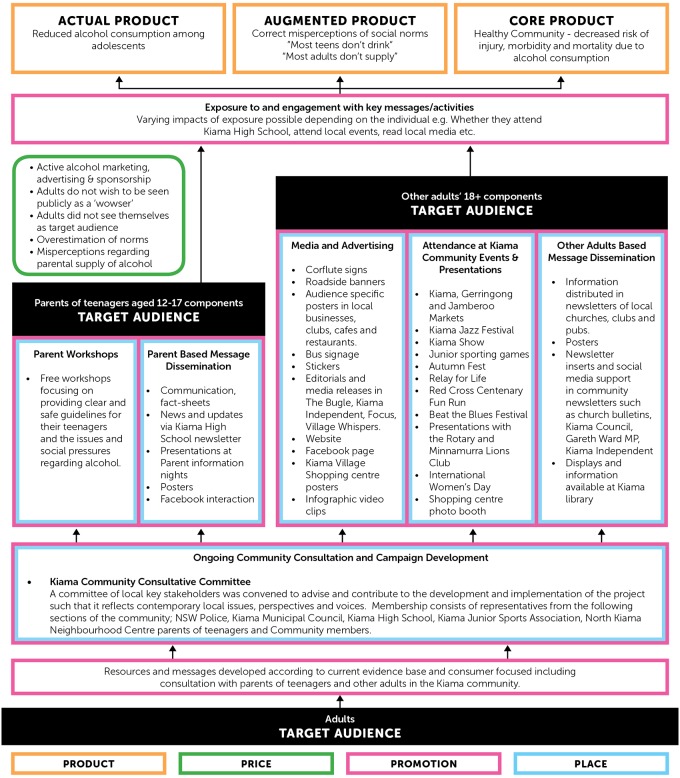
Project Logic Model—Adult Target Audience.

First, a detailed program plan was devised, incorporating environmental aspects (e.g.: available media outlets, spokespeople, the role of local government, schools etc), a community calendar of events, available resources and realistic timelines. Project logic models, represented by Figs 4 and 5 were consolidated for both the adult and adolescent target populations to ensure clarity and purpose. Secondly, a ‘community consultative committee’ with representatives of key local stakeholders was established to facilitate a collaboration between researchers and the community. This committee’s clear purpose was to inform and reflect on appropriate project strategies using local knowledge, networks and opportunities. The committee met bi-monthly. Thirdly, in order to measure the interim successes or challenges of implementation strategies, record sheets were developed and used to record the volume and nature of distribution of information and merchandise; and key questions were devised to be used by project staff to ‘open conversations’ and ascertain the reach, credibility and understanding of project messages by the target audiences.

Finally, twelve months after the launch of the intervention, in order to assess the reach of the intervention and to provide data for the subsequent phases, the CATI provider re-contacted those who had agreed to follow-up (n = 530, 86.9% of the baseline sample). A total of 397 (74.7%) were able to be contacted and completed the follow-up survey. Demographic details of the sample are shown in Table 2; those who participated in Wave 2 did not differ on these variables from the initial baseline sample.

**Table 2:** Respondent demographics.

	Full baseline sample(N = 610)	Study sample (N = 397)	Chi-square	P
Children in family				
Aged 0–11	24.9%	27.5%	0.81	0.37
Aged 12–17	28.9%	33.8%	2.71	0.11
Aged18+	41.8%	42.8%	0.10	0.79
Age				
<40	28.0%	25.7%	0.80	0.67
40–54	50.8%	53.4%		
55+	21.1%	20.9%		
Country of birth				
Australia	88.4%	87.7%	0.11	0.77
Other	11.6%	12.3%		
Household structure				
married/defacto no kids	13.4%	12.4%	1.19	.55
married/defacto with kids	64.3%	68.2%		
One parent	8.4%	7.1%		
Employment status				
Full-time	42.6%	44.6%	1.12	.77
Part-time	26.4%	27.2%		
Unemployed, student, other	13.8%	13.3%		
Not in labour force	17.2%	14.9%		
Education level				
Yr 10 or less	12.6%	9.6%	1.82	0.61
Yr 11–12	13.6%	13.8%		
Cert/dip/trade	30.0%	30.0%		
Uni	43.8%	44.3%		
Religion				
Anglican	18.9%	20.7%	0.59	0.90
Catholic	23.1%	23.2%		
No religion	31.0%	30.5%		
Other	27.0%	25.6%		

Continuous variables (perceived percentage of young people who drink alcohol; acceptable age at which a young person can try alcohol) were compared across wave 1 (baseline) and wave 2 (follow-up) with repeated measures t-tests. There was a small amount of missing data for the question regarding what percentage of 14 year olds drink alcohol. In wave 1 n = 24 had missing data (6%) and wave two there were 22 missing cases (5.5%). The other two questions had a maximum of 14 missing cases (3.5%). Cases with missing data were excluded from the analysis. For the questions regarding acceptable age to consume alcohol missing data ranged from no cases to 14 cases (3.5%), again, cases without data were excluded from the analysis. The Likert options of very unacceptable and unacceptable were combined for the analysis regarding acceptable behavior with regard to the supply of alcohol to minors. There were no missing data for these questions resulting in a sample of n = 397. A z-test was used to compare the proportions of respondents who classified the behavior as unacceptable at wave 1 and wave 2.

## Results—Process Evaluation

### Campaign Dissemination

During the two years of the intervention, we put up more than 2,600 advertising posters, 8 banners, and 200 corflutes. We distributed 5,000 booklets (easy-to-read facts on underage drinking and tips for parents); and a further 20,000 pieces of collateral, including hacky sacks, highlighter pens, glow in the dark wrist bands, shopping bags, wallets, pens, magnets, post it notes, and disposable coffee cups (provided to coffee vendors at markets/events).

We purchased 23 paid media spots (advertisements in local newspapers), and gained 15 earned media spots (editorials on the intervention, opportunities for community engagement, activities and outcomes). Campaign staff attended 65 community events, engaging in conversations with members of the local community and distributing promotional materials.

Our website attracted 3,640 visits from 2,675 unique visitors; with 8,596 page views (the most popular page was the tab for downloadable fact sheets). The campaign’s Facebook page reached 842 likes by the end of the campaign, with evidence of a far greater reach of posts on the page.

### Campaign Awareness

Of the 397 respondents who completed the CATI survey, 85.6% (n = 340) reported that they had seen or heard any messages, information or events in their local community about underage drinking in the last 12 months. The most common places people recalled seeing this information were signs on fences around town (66.8%) and posters in shops, library, cafes etc (45.9%). Our ongoing environmental scan identified no other messages about underage drinking in either of these locations during the period of our campaign. Almost one in five (17.6%) reported seeing such information in ads and 8.8% in editorial items in the local newspaper; we can confirm that the former were references to our campaign as there were no other advertisers on this topic during this time period, but the latter may have been related to our campaign or other issues. Similarly, the 11.5% who saw messages at the local high school can definitely be attributed to our campaign, which the school actively partnered in.

In response to the open-ended question “what is the main message you remember”, over half (53.5%, n = 182) recalled the correct tagline “Kiama Doesn’t Support Underage Drinking.” A further 8.5% recalled the call to action from the outdoor advertising “Can a Community Stop Underage Drinking?” and 6.2% (n = 21) the underlying message of the campaign (e.g., “We need to make it okay for young people not to drink”).

It is noteworthy that the initial campaign tagline, which was developed based on the stated preferences for high-fear messages expressed by participants in the formative and message testing phases was only recalled by seven respondents (2.1%). This would be in part due to the time lag between the end of that phase and the timing of the survey, but also supports the decision to progress with the social norms messaging.

### Preliminary Evidence of Impact

The primary aim of the 12-month follow up survey was to provide data for the subsequent intervention phases; that is we were hoping to identify one or two small changes in community knowledge and/or attitudes that could be used to reinforce the messages. The magnitude of the changes provided additional motivation for the project team and the community.

There was a significant drop in the perceived percentage of young people who drink alcohol, for all three age points (see Table 3). These changes were between six and seven percentage points; for example, the average perceived proportion of 16-year-olds who are drinkers decreased from 53.1% to 45.7% (p < 0.001). Perceptions of the prevalence of youth drinking declined significantly both among those who were the parents of 12-to-17-year-olds and those who did not have children in this age group.

**Table 3:** Perceived proportion of young people in the community who are drinkers.

	All			Parents^1^			Non-parents^1^		
	N	Baseline	12-months	n	Baseline	12-months	n	Baseline	12-months
18 –year-olds	381	80.7	73.2***	145	80.9	74.6***	236	80.6	72.3***
16-year-olds	378	53.1	45.7***	146	49.6	45.6*	232	55.3	45.8***
14-year-olds	355	25.1	19.3***	137	22.0	16.9**	218	27.0	20.8***

^1^ Parents = those who are the parent of a 12-17-year-old; Non-parents = those who are not the parent of a child in this age group

* = p < 0.05

** = p < .01

*** = p < .0001

There was an increase in the average age at which people believe it is acceptable for young people to have a sip or taste of alcohol (from 16.1 years to 16.5 years, p < 0.001). This effect was stronger among those who were not the parents of a child aged 12-to-17-years-old (.56 of a year) than those who were parents of children in this age group (.33 of a year), but was statistically significant for both groups.

Among parents, perceptions of the acceptability of supply of alcohol to a 16-year-old by parents, and by other adults, in three contexts (at home, at a supervised party, and an unsupervised party) showed changes in the intended direction. However, this was only statistically significant for a parent providing alcohol for a 16-year-old to drink at home (from 46.6% to 58.8%, p = 0.036); and for a parent providing alcohol for a 16-year-old to drink away from home at a supervised party (from 59.5% to 71.6%, p = 0.027). There were no significant changes in perceptions among non-parents. However, it is important to note that at baseline parents considered each of these behaviors to be more widely acceptable in the community than did non-parents, and the shift in parents’ perceptions between baseline and 12-months brought their perceptions into alignment with those of non-parents (i.e., the broader community).

## Discussion

While there is widespread recognition that children’s and adolescents’ drinking is strongly influenced by social norms, and increasing evidence that parents’ and other adults’ decisions to provide alcohol are likewise influenced, there are surprisingly few published papers on interventions that seek to address these norms at a community level [24].

The baseline research that guided this intervention confirmed that both adolescents and adults misperceive community norms relating to underage drinking and the supply of alcohol to minors. The formative research showed that adolescents and adults perceive that current messages about underage drinking target ‘other’ people; and that parents’ decisions to provide alcohol are influenced by complex and competing pressures.

The process evaluation data provides evidence that a community-based social norms message delivered within a social marketing framework can achieve high cut-through among the multitude of messages that people are exposed to in an increasingly busy and media-risk world. The majority of respondents surveyed 12 months after commencement of the intervention were aware of its existence, and over half could correctly recall the current message tagline.

The process evaluation also provided preliminary evidence that a whole-of-community social marketing intervention may be able to address misperceptions about social norms, as evidenced by the reductions in the perceived proportion of teen drinkers in the community among both parents and community members. We also saw significant increases in parents’ perceptions of the proportion of their community who believe it is unacceptable for a parent to provide their 16-year-old with alcohol. While this data was only collected in the intervention community, and the lack of a control group means we cannot conclusively rule out the possibility that the changes were part of broader social changes, their magnitude in such a short space of time augurs well for the capacity of such an intervention to, at a minimum, start a conversation with the community.

This intervention was conducted in a single regional community, and thus the findings may not be generalizable to other regions or countries. However, these findings provide preliminary evidence that a social marketing approach to social norms interventions may allow us to extend the effectiveness SNA beyond correcting misperceptions of social norms in the immediate peer group (as has been demonstrated to be effective in previous studies) to correcting misperceptions of broader societal norms.

Australia, like many countries, is currently observing declines in youth alcohol consumption—reversing the trend of the last two decades. While the reasons for this change are not clearly understood, it is timely for communities and governments to build on this positive trend by providing environments that support not drinking as an acceptable and normative behavior for adolescents. Social marketing can play a key role in achieving this outcome.
